# The embryonic genes Dkk3, Hoxd8, Hoxd9 and Tbx1 identify muscle types in a diet-independent and fiber-type unrelated way

**DOI:** 10.1186/1471-2164-11-176

**Published:** 2010-03-15

**Authors:** Janneke de Wilde, Martijn FM Hulshof, Mark V Boekschoten, Philip de Groot, Egbert Smit, Edwin CM Mariman

**Affiliations:** 1 Top Institute Food and Nutrition, Nutrigenomics Consortium, Wageningen, the Netherlands; 2 Department of Human Biology, Nutrition and Toxicology Research Institute Maastricht (NUTRIM), Maastricht University, Maastricht, the Netherlands; 3 Nutrition, Metabolism and Genomics group, Wageningen University, Wageningen, the Netherlands

## Abstract

**Background:**

The mouse skeletal muscle is composed of four distinct fiber types that differ in contractile function, number of mitochondria and metabolism. Every muscle type has a specific composition and distribution of the four fiber types. To find novel genes involved in specifying muscle types, we used microarray analysis to compare the gastrocnemius with the quadriceps from mice fed a low fat diet (LFD) or high fat diet (HFD) for 8 weeks. Additional qPCR analysis were performed in the gastrocnemius, quadriceps and soleus muscle from mice fed an LFD or HFD for 20 weeks.

**Results:**

In mice fed the 8-week LFD 162 genes were differentially expressed in the gastrocnemius *vs*. the quadriceps. Genes with the strongest differences in expression were markers for oxidative fiber types (e.g. *Tnni1*) and genes which are known to be involved in embryogenesis (*Dkk3*, *Hoxd8*,*Hoxd9 *and *Tbx1*). Also *Dkk2, Hoxa5, Hoxa10, Hoxc9, Hoxc10, Hoxc6 *and *Tbx15 *were detectably, but not differentially expressed in adult muscle tissue. Expression of differentially expressed genes was not influenced by an 8-week or 20-week HFD. Comparing gastrocnemius, quadriceps and soleus, expression of *Hoxd8 *and *Hoxd9 *was not related with expression of markers for the four different fiber types. We found that the expression of both *Hoxd8 *and *Hoxd9 *was much higher in the gastrocnemius than in the quadriceps or soleus, whereas the expression of *Dkk3 *was high in quadriceps, but low in both gastrocnemius and soleus. Finally, expression of *Tbx1 *was high in quadriceps, intermediate in soleus and low in gastrocnemius.

**Conclusions:**

We found that genes from the Dkk family, Hox family and Tbx family are detectably expressed in adult mouse muscle. Interestingly, expression of *Dkk3*, *Hoxd8, Hoxd9 *and *Tbx1 *was highly different between gastrocnemius, quadriceps and soleus. In fact, every muscle type showed a unique combination of expression of these four genes which was not influenced by diet. Altogether, we conclude that genes important for embryogenesis identify mouse muscle types in a diet-independent and fiber type-unrelated manner.

## Background

The mouse skeletal muscle is composed of four distinct fiber types, i.e., the type I, IIa, IIx and IIb fibers that differ in respect to contractile function, the number of mitochondria and metabolism. The slow type I fibers can sustain prolonged low power work, contain more mitochondria and exhibit higher rates of fat oxidative metabolism. The fast type IIx and IIb fibers are adapted to brief and intense contractions, contain fewer mitochondria and generate energy mainly through glycolysis. The type IIa fibers exhibit an intermediate contractile function and are oxidoglycolytic [1-4]. Every muscle type has a specific composition and distribution of the four different fiber types. The soleus contains the highest number of type I fibers, whereas both the quadriceps and gastrocnemius are muscle groups that contain predominantly type II fibers [5,6]. However, varying percentages of type I fibers ranging from 0-45% for the quadriceps and 1-8% for the gastrocnemius are reported [7].

Numerous genes have been identified playing a role in the generation of more oxidative muscle types [6,8-13]. The most extensively studied gene is calcineurin (*CnA*). In the C2C12 cell line, *CnA *up-regulates the mRNA levels of genes that are markers for the slow fiber types. Additionally, studies with *CnA *transgenic mice have reported an increase of oxidative fibers in the skeletal muscle [8,9]. *PGC1α *is another well-established factor that induces remodeling of skeletal muscle fiber type composition. Lin et al. [10] showed that when *PGC1α *is expressed at physiological levels in muscle groups normally rich in type II fibers a fiber type conversion occurs from type II fibers to type I fibers [10]. In line with this, Mortensen et al. [11] showed that *PGC1α *overexpression in primary rat skeletal muscle cells induces a switch towards a more oxidative fiber type. Finally, muscle-specific overexpression of *PPARδ *resulted in the increase of the number of oxidative fibers and in higher expression levels of several markers of oxidative metabolism [6,12].

The first aim of the present study was to find novel genes that may play a role in specifying muscle types. Therefore we compared gene expression profiles of the gastrocnemius with the quadriceps at the level of the whole-transcriptome. Functional implications were assessed by the analyses of predefined gene sets based on Gene Ontology, biochemical, metabolic and signaling pathways. Recently, we studied the effects of a short-term high fat diet (HFD) on skeletal muscle gene and protein expression. Since both gene and protein levels of markers of the more oxidative fiber types were increased in the quadriceps of HFD mice we hypothesized that an HFD can induce a conversion towards a more oxidative fiber type via a transcriptional mechanism [14]. The second aim of the present study therefore was to explore the diet-sensitivity of genes that are involved in the determination of muscle types. We compared expression patterns of genes that are differentially expressed between muscle types in mice fed a low fat diet (LFD) with mice fed an HFD.

## Results

### Microarray analysis shows that the majority of genes involved in the determination of muscle types are not influenced by diet

**Figure 1:**
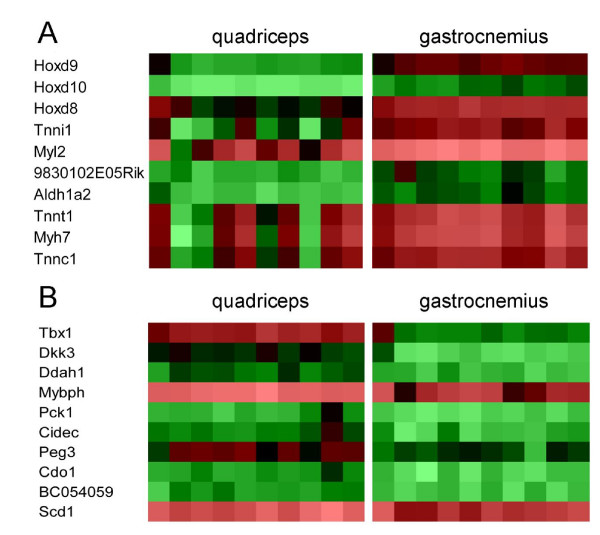
**Heatmap of the ten genes with the strongest difference in expression between gastrocnemius and quadriceps**. Heatmap of the log-transformed microarray signal intensity values of the ten genes that showed the strongest increased (A) and decreased (B) expression level in the gastrocnemius as compared to the quadriceps of 10 LFD mice. The heatmap was generated by using GeneMaths XT software. Signal intensities are shown by a color range; bright red, black, and bright green represent high, average, and low levels of gene expression, respectively.

To obtain more insight in genes involved in specifying muscle types we compared the gastrocnemius transcriptome with the quadriceps transcriptome of 8-week LFD mice. When using the criteria of a fold change > 1.3 and an FDR < 0.05, we found 91 genes with an increased expression and 71 genes with a decreased expression in the gastrocnemius as compared to the quadriceps. The ten genes with the strongest increased and decreased expression levels are shown in Table 1 and Fig. 1. The ten genes with the strongest increased expression included markers of oxidative fiber types (*Tnnc1, Tnni1, Tnnt1 and Myh7*), but also genes from the Hox family (*Hoxd8, Hoxd9 and Hoxd10*). In fact, *Hoxd8, Hoxd9 *and *Hoxd10 *were the genes with the strongest increased expression in the gastrocnemius. The ten genes with the strongest decreased expression contained a variety of genes such as *Scd1 *and *Cidec*, which are involved in lipogenesis, and *Pck1*, which plays a role in gluconeogenesis, but also the *Dkk3 *and *Tbx1 *genes, which are important during embryogenesis. A complete list of all differentially expressed genes is available in supplement 1. qPCR analyses were performed for 13 genes validating the microarray results (supplement 2).

**Table 1:** Ten genes that showed the strongest increased or decreased expression in gastrocnemius compared to quadriceps

*probe set*	*gene name*	*gene description*	*Accession (EntrezID)*	*Fold change*	*FDR*
Strongest up-regulated genes					
15438_at	Hoxd9	homeo box D9	15438	7.70	0.000
15430_at	Hoxd10	homeo box D10	15430	7.36	0.000
15437_at	Hoxd8	homeo box D8	15437	4.78	0.000
21952_at	Tnni1	troponin I, skeletal, slow 1	21952	4.46	0.005
17906_at	Myl2	myosin, light polypeptide 2, regulatory, cardiac, slow	17906	3.89	0.004
277898_at	9830102E05Rik	RIKEN cDNA 9830102E05 gene	277898	3.69	0.002
19378_at	Aldh1a2	aldehyde dehydrogenase family 1, subfamily A2	19378	3.61	0.000
21955_at	Tnnt1	troponin T1, skeletal, slow	21955	3.56	0.024
140781_at	Myh7	myosin, heavy polypeptide 7, cardiac muscle, beta	140781	3.26	0.039
21924_at	Tnnc1	troponin C, cardiac/slow skeletal	21924	3.05	0.020
Strongest down-regulated genes					
21380_at	Tbx1	T-box 1	21380	-7.27	0.000
50781_at	Dkk3	dickkopf homolog 3 (Xenopus laevis)	50781	-5.40	0.000
69219_at	Ddah1	dimethylarginine dimethylaminohydrolase 1	69219	-2.91	0.000
53311_at	Mybph	myosin binding protein H	53311	-2.66	0.002
18534_at	Pck1	phosphoenolpyruvate carboxykinase 1, cytosolic	18534	-2.62	0.015
14311_at	Cidec	cell death-inducing DFFA-like effector c	14311	-2.57	0.009
18616_at	Peg3	paternally expressed 3	18616	-2.49	0.005
12583_at	Cdo1	cysteine dioxygenase 1, cytosolic	12583	-2.45	0.004
246747_at	BC054059	cDNA sequence BC054059	246747	-1.96	0.010
20249_at	Scd1	stearoyl-Coenzyme A desaturase 1	20249	-1.79	0.018

By microarray analysis we found 161 that were differentially expressed in the gastrocnemius as compared with the quadriceps from mice fed an 8-week LFD. This table shows the ten strongest up-regulated and ten strongest down-regulated genes in the gastrocnemius as compared with the quadriceps (*n *= 10).

To relate changes in gene expression to biological changes we applied ErmineJ and GSEA. By using Ermine J we identified 68 GO classes that were overrepresented in the gastrocnemius (Table 2). These overrepresented GO classes were mainly descriptors for contraction, morphogenesis, remodeling and development. Other overrepresented GO classes included descriptors for calcium homeostasis, immune function and metabolic processes. A parallel GSEA was used to identify up- or down-regulated processes. GSEA revealed that ten gene sets were down-regulated including three gene sets related to the cell cycle, two gene sets describing amino acid metabolism and two gene sets corresponding to immune function. Only the gene sets "striated muscle contraction" and "ribosome" were up-regulated in the gastrocnemius as compared with the quadriceps. Among the core-enriched genes in the gene set "striated muscle contraction" were genes that are markers for oxidative fiber types, whereas all core-enriched genes in the gene set "ribosome" were ribosomal proteins which are involved in translation (Table 3).

**Table 2:** Overrepresented Gene Ontology classes in the gastrocnemius as compared to the quadriceps

*GO ID*	*GO class*	*N*	*Raw Score*	*FDR*
GO:0001974	blood vessel remodeling	15	1.99	2.39E-10
GO:0006776	vitamin A metabolic process	15	1.92	9.95E-11
GO:0051705	behavioral interaction between organisms	18	1.63	3.89E-04
GO:0006775	fat-soluble vitamin metabolic process	20	1.57	4.17E-04
GO:0048705	skeletal morphogenesis	17	1.41	8.14E-04
GO:0009855	determination of bilateral symmetry	31	1.30	5.97E-11
GO:0003007	heart morphogenesis	29	1.28	8.53E-11
GO:0014033	neural crest cell differentiation	31	1.28	3.98E-10
GO:0006936	muscle contraction	69	1.19	1.49E-10
GO:0006937	regulation of muscle contraction	25	1.19	7.35E-04
GO:0043010	camera-type eye development	56	1.14	7.02E-11
GO:0007368	determination of left/right symmetry	30	1.14	8.02E-04
GO:0048762	mesenchymal cell differentiation	38	1.13	9.19E-11
GO:0008016	regulation of heart contraction	34	1.13	3.23E-04
GO:0060047	heart contraction	41	1.11	1.09E-10
GO:0007498	mesoderm development	44	1.06	1.33E-10
GO:0001654	eye development	82	1.03	7.46E-11
GO:0048592	eye morphogenesis	37	1.02	6.97E-04
GO:0006766	vitamin metabolic process	56	1.01	6.28E-11
GO:0009952	anterior/posterior pattern formation	86	0.96	4.59E-11
GO:0016055	Wnt receptor signaling pathway	107	0.94	4.98E-11
GO:0042445	hormone metabolic process	71	0.92	1.71E-04
GO:0048771	tissue remodeling	104	0.92	1.71E-10
GO:0006875	cellular metal ion homeostasis	57	0.92	2.99E-04
GO:0048637	skeletal muscle development	61	0.92	7.96E-11
GO:0006959	humoral immune response	52	0.92	7.46E-04
GO:0007519	striated muscle development	103	0.91	1.19E-10
GO:0055065	metal ion homeostasis	58	0.91	1.99E-04
GO:0030324	lung development	59	0.89	4.07E-04
GO:0007178	transmembrane receptor protein serine/threonine kinase signaling pathway	67	0.89	1.93E-04
GO:0006575	amino acid derivative metabolic process	70	0.88	5.69E-11
GO:0030323	respiratory tube development	60	0.88	4.78E-04
GO:0007229	integrin-mediated signaling pathway	74	0.88	2.91E-04
GO:0048469	cell maturation	66	0.87	4.68E-04
GO:0006817	phosphate transport	61	0.86	2.84E-04
GO:0055074	calcium ion homeostasis	53	0.86	7.58E-04
GO:0045786	negative regulation of progression through cell cycle	101	0.85	5.19E-11
GO:0048732	gland development	63	0.85	6.85E-04
GO:0044271	nitrogen compound biosynthetic process	61	0.84	3.98E-04
GO:0030003	cellular cation homeostasis	87	0.84	1.99E-10
GO:0021700	developmental maturation	79	0.84	2.21E-04
GO:0016311	dephosphorylation	105	0.83	5.97E-10
GO:0055080	cation homeostasis	88	0.83	2.13E-04
GO:0055082	cellular chemical homeostasis	90	0.82	5.43E-11
GO:0006470	protein amino acid dephosphorylation	93	0.82	6.63E-11
GO:0050778	positive regulation of immune response	75	0.81	3.32E-04
GO:0051240	positive regulation of multicellular organismal process	94	0.81	1.76E-04
GO:0008015	circulation	84	0.81	5.43E-04
GO:0050776	regulation of immune response	88	0.80	1.87E-04
GO:0002684	positive regulation of immune system process	77	0.80	4.59E-04
GO:0050801	ion homeostasis	103	0.79	2.99E-10
GO:0002682	regulation of immune system process	90	0.79	2.06E-04
GO:0001503	ossification	80	0.79	4.51E-04
GO:0045165	cell fate commitment	94	0.78	4.42E-04
GO:0015674	di-, tri-valent inorganic cation transport	112	0.78	1.19E-09
GO:0031214	biomineral formation	81	0.78	7.21E-04
GO:0046849	bone remodeling	89	0.78	3.81E-04
GO:0008361	regulation of cell size	91	0.77	4.78E-11
GO:0030005	cellular di-, tri-valent inorganic cation homeostasis	81	0.77	7.08E-04
GO:0043062	extracellular structure organization and biogenesis	85	0.77	6.28E-04
GO:0009968	negative regulation of signal transduction	81	0.77	6.74E-04
GO:0040008	regulation of growth	117	0.77	1.81E-04
GO:0006816	calcium ion transport	85	0.75	8.78E-04
GO:0002009	morphogenesis of an epithelium	103	0.73	4.87E-04
GO:0002252	immune effector process	106	0.73	6.40E-04
GO:0043549	regulation of kinase activity	120	0.72	3.14E-04
GO:0045859	regulation of protein kinase activity	115	0.72	3.73E-04
GO:0051338	regulation of transferase activity	121	0.72	3.06E-04

ErmineJ was used to identify significantly overrepresented GO classes in the gastrocnemius as compared with the quadriceps from LFD mice (*n *= 10). For the concept biological process we selected GO classes with a FDR < 0.001. For this analysis only classes containing 8 through 125 genes were taken into account. N, number of genes in GO class

**Table 3:** Changed gene sets in the gastrocnemius versus quadriceps.

	*N*	*ES*	*NES*	*FDR*
up-regulated cellular processes				
				
Striated muscle contraction^1^	41	0.68	2.37	0.000
Ribosome^2^	20	0.71	2.05	0.005
down-regulated cellular processes				
				
Tissues, muscle, fat, bone and connective 2^1^	47	-0.57	-2.14	0.003
Cell cycle^1^	85	-0.52	-2.14	0.003
CTCF first multivalent nuclear factor^3^	17	-0.71	-2.07	0.006
Valine, leucine and isoleucine degradation^2^	40	-0.59	-2.16	0.006
Cell cycle G1 to S control reactome^2^	69	-0.49	-2.01	0.013
Antigen processing and presentation^2^	51	-0.52	-2.01	0.014
Mets affect on macrophage differentiation^3^	16	-0.69	-1.97	0.014
Amino acid metabolism^1^	46	-0.52	-1.95	0.016
Porphyrin and Chlorophyll metabolism^2^	19	-0.64	-1.88	0.033
DNA replication reactome^1^	40	-0.51	-1.86	0.038

GSEA was applied to identify up-regulated and down-regulated processes in the gastrocnemius as compared with the quadriceps in LFD mice. Presented are regulated processes with a false discovery rate (FDR) < 0.05. An FDR was calculated to adjust for multiple hypothesis testing. Sources of the gene sets: ^1^GenMAPP, ^2^KEGG, ^3^Biocarta. N, number of genes; ES, enrichment score for the gene set, that reflects the degree to which a gene set is overrepresented at the top or bottom of the ranked list; FDR, NES, normalized enrichment score, that is, the normalized ES to account for the size of the set.

The diet-sensitivity of differentially expressed genes, overrepresented GO classes and regulated gene sets was studied by comparing the gastrocnemius with the quadriceps under HFD conditions (supplement 3). In the HFD mice we found 215 differentially expressed genes in the gastrocnemius as compared to the quadriceps. A total of 128 genes was overlapping with the differentially expressed genes in the LFD mice. Interestingly, these overlapping genes were all regulated in the same direction with a comparable fold change. A total of 87 genes was only differentially expressed in HFD mice, whereas 34 genes were differentially expressed in LFD mice suggesting that these genes are diet-dependent. Under HFD conditions, ErmineJ revealed that 193 GO classes were overrepresented in the gastrocnemius as compared to the quadriceps. A total of 67 GO classes showed an overlap with the overrepresented GO classes under LFD conditions. The other overrepresented GO classes corresponded also to contraction, morphogenesis, remodeling, development, calcium homeostasis, immune function and metabolic processes. GSEA showed that 11 gene sets were down-regulated including five gene sets describing cell cycle and four gene sets related to amino acid metabolism. Only one gene set "striated muscle contraction" was up-regulated in the gastrocnemius *vs*. the quadriceps. A total of six gene sets (five down-regulated and one up-regulated) were overlapping between the LFD and HFD condition.

To summarize, we showed that a considerable number of genes is differentially expressed in the gastrocnemius as compared with the quadriceps. The genes with the strongest regulations were markers for oxidative fiber types (*Tnnc1, Tnni1, Tnnt1 *and *Myh7*) and genes which are known to be involved in embryogenesis (*Dkk3, Hoxd8, Hoxd9, Hoxd10 *and *Tbx1*). The differentially expressed genes corresponded to a variety of process including morphogenesis and contraction, but also amino acid metabolism, cell cycle and immune function. Finally, we found more differentially expressed genes and more overrepresented GO classes under HFD conditions than under LFD conditions. However, this did not result in the identification of other regulated processes indicating that diet has only a very small effect on the difference between gastrocnemius and quadriceps.

### Numerous genes involved in embryogenesis are detectably expressed in adult mouse muscle tissue

**Figure 2:**
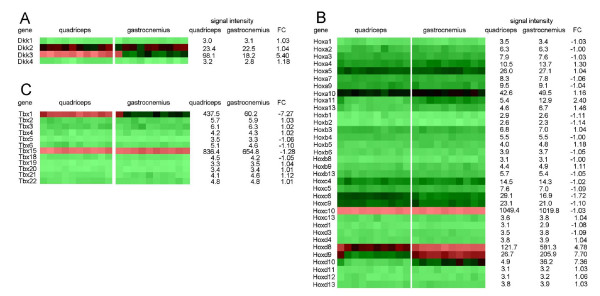
**Heatmap of Dkk genes, Hox genes and Tbx genes in gastrocnemius and quadriceps**. Heatmap of the log-transformed microarray signal intensity values of (A) Dkk genes, (B) Hox genes and (C) Tbx genes in the quadriceps and the gastrocnemius of 10 LFD mice. A heatmap was generated by using GeneMaths XT software. Signal intensities are shown by a color range; bright red, black, and bright green represent high, average, and low levels of gene expression, respectively. Values are means (*n *= 10)

One of the most interesting findings of the comparison between gastrocnemius and quadriceps was the fact that genes of the Dkk family (*Dkk3*), Hox family (*Hoxd8, Hoxd9 *and *Hoxd10*) and Tbx family (*Tbx1*) were detectably expressed in adult mouse muscle tissue. To find out if also other genes from these three gene families are detectably expressed in adult mouse muscle tissue we analyzed the microarray signal intensities of all members, which were present on the microarray, in the gastrocnemius and quadriceps. An average microarray signal intensity > 20 was used as cut off. Fig. 2A shows that four members of the Dkk gene family were present on the microarray. In addition to *Dkk3*, we found that also *Dkk2 *was detectably expressed in both gastrocnemius and quadriceps (22.5 vs. 23.4 in gastrocnemius vs. quadriceps). From the Hox family 34 members were present on the microarray. Eight genes of the Hox family, including *Hoxd8, Hoxd9 *and *Hoxd10*, were expressed in adult muscle tissue. *Hoxa5, Hoxa10, Hoxc9, Hoxc10, Hoxd8 *and *Hoxd9 *were detectably expressed in both the gastrocnemius and quadriceps. Furthermore, *Hoxc6 *was detectably expressed in the quadriceps and not in the gastrocnemius (29.1 versus 16.9), whereas *Hoxd10 *was detectably expressed in the gastrocnemius and not in the quadriceps (36.2 versus 4.9). The gene with the highest expression level was identified as the *Hoxc10 *gene (Fig. 2B). Fig. 2C shows that from the *Tbx *family 12 members were present on the microarray. Additionally to *Tbx1*, we found that *Tbx15 *was highly expressed in the gastrocnemius as well as in the quadriceps (654.8 vs. 836.4, respectively). Altogether, these results show that some genes that are known to play an important role in embryogenesis are also detectably expressed in adult mouse muscle tissue.

### The Tbx1 gene is a muscle-type specific marker

**Figure 3:**
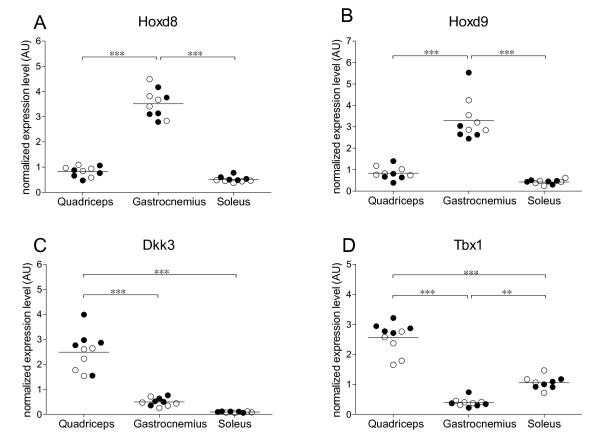
**Expression of *Hoxd8, Hoxd9, Dkk3 *and *Tbx1 *in quadriceps, gastrocnemius and soleus**. Gene expression levels of (A) *Hoxd8*, (B) *Hoxd9*, (C) *Dkk3 *and (D) *Tbx1 *in quadriceps, gastrocnemius and soleus of 20-week LFD mice and 20-week HFD mice. Black dots represent the 20-week LFD mice (*n *= 5); open dots represent the 20-week HFD mice (*n *= 5), lines represent grand mean values. *** Indicate significant differences with *p *< 0.001 obtained with two-way ANOVA

*Dkk3, Hoxd8, Hoxd9 *and *Tbx1 *were relatively high and differentially expressed in the gastrocnemius as compared to the quadriceps of mice fed an 8-week LFD. To extrapolate our result to a third muscle type we analyzed the expression level of these genes in quadriceps, gastrocnemius and soleus muscle of 20-week LFD mice and 20-week HFD mice. None of the genes was significantly influenced by diet which is in line with the earlier described observations (Fig. 3A-D). *Hoxd8 *and *Hoxd9 *were highly expressed in the gastrocnemius, but low gene expression levels were observed in the quadriceps and soleus (Fig. 3A-B). *Dkk3 *was significantly higher expressed in the quadriceps than in the gastrocnemius and soleus. Although not significantly, *Dkk3 *gene expression was lower in the soleus than in the gastrocnemius (Fig. 3C). The highest expression levels of the *Tbx1 *gene was found in the quadriceps. Intermediate expression levels were measured in the soleus and the lowest expression level was found in the gastrocnemius. The *Tbx1 *expression levels between the three muscle types were all significantly different (Fig. 3D).

### Gene expression patterns of Hoxd8 and Hoxd9 do not correlate with expression patterns of markers for the different fiber types

**Figure 4:**
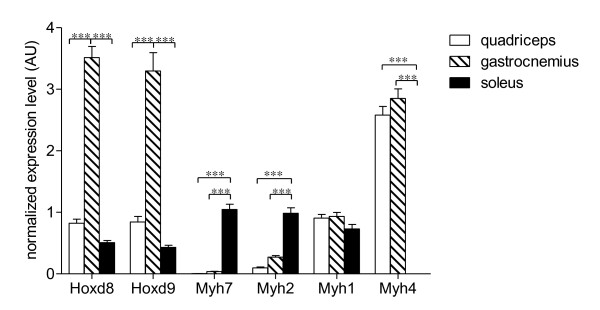
**Expression of *Hoxd8, Hoxd9, Myh7, Myh2, Myh1 *and *Myh4 *in quadriceps, gastrocnemius and soleus**. Gene expression levels of *Hoxd8, Hoxd9*, *Myh7*, *Myh2*, *Myh1 *and *Myh4 *in quadriceps, gastrocnemius and soleus of 20-week LFD and HFD mice. The genes *Myh7, Myh2, Myh1 *and *Myh4 *are markers for type I, IIa, IIx and IIb fiber types, respectively. White bars, dashed bars and black bars represent quadriceps, gastrocnemius and soleus, respectively. Bars represent grand mean values of 20-week LFD mice and 20-week HFD mice. *** Indicate significant differences with *p *< 0.001 obtained with two-way ANOVA

We found that markers for the oxidative fiber types as well as members of the Hoxd gene cluster were higher expressed in the gastrocnemius than in the quadriceps. To find out if these Hoxd genes play a role in specifying more oxidative muscle types we compared genes expression levels of *Hoxd8, Hoxd9 *and markers for different fiber types (type I: *Myh7*; type IIa: *Myh2*; type IIx: *Myh1 *and type IIb: *Myh4*) in quadriceps, gastrocnemius and soleus muscle of 20-week LFD mice and 20-week HFD mice. Expression levels of markers for the different fiber types were not affected by diet. Whereas *Hoxd8 *and *Hoxd9 *showed increased expression in the gastrocnemius as compared to quadriceps and soleus, expression levels of *Myh7 *was significantly higher in the soleus than in the quadriceps and gastrocnemius, respectively. Also the *Myh2 *expression level was higher in the soleus than in the quadriceps and gastrocnemius. For both *Myh7 *and *Myh2 *no differences between the quadriceps and the gastrocnemius were observed. *Myh1 *gene expression levels were comparable between the three muscle types. Finally, the expression level of the *Myh4 *gene was significantly lower in the soleus than in the quadriceps and gastrocnemius, respectively. *Myh4 *gene expression levels were similar in the quadriceps and gastrocnemius (Fig. 4). Altogether, the expression patterns of *Hoxd8 *and *Hoxd9 *did not correspond with the expression patterns of one of the markers for the different fiber types.

## Discussion

In the present study we searched for novel genes that are involved in specifying muscle types. Additionally, we studied the diet-sensitivity of differentially expressed genes. By comparing the gastrocnemius with the quadriceps we identified 162 differentially expressed genes corresponding to a variety of biological processes. Especially processes involved in cell cycle, contraction, development, differentiation, morphogenesis and remodeling were differentially regulated in the gastrocnemius vs. the quadriceps. Both the gastrocnemius and the quadriceps are muscle groups that consist of predominantly type II fibers. In the present study we found that *Myh7, Tnnc1, Tnni1 *and *Tnnt1*, which are all markers for the more oxidative fiber types, are strongly up-regulated in the gastrocnemius as compared to the quadriceps. Western blotting showed that protein levels of myosin heavy chain, slow fiber type protein (marker for the oxidative type I fibers) were similar between the gastrocnemius and the quadriceps, whereas the protein level of Myh2 (marker for the oxidoglycolytic type IIA fibers) was higher in the gastrocnemius than in the quadriceps (data not shown). Therefore, we propose that in this study the gastrocnemius had a more oxidative character than the quadriceps.

Recently, we showed that a 4-week HFD intervention results in increased gene expression and protein levels of markers for the oxidative fiber types in the quadriceps of mice. These findings suggested that the skeletal muscle can adapt to an increased lipid load by inducing a switch to a more oxidative phenotype [14]. Although the adult skeletal muscle is known to have the capacity to adapt to functional demands including exercise, hormones, innervation and mechanical loading [15,16], a link between HFD and a switch of fiber type was not yet described. However, in the present study we showed that an 8-week HFD has none or only minor effects on expression levels of genes that play a role in specifying different muscle types including the markers for oxidative fiber types. Therefore we speculate that the up-regulation of markers for the oxidative fiber types is an early response of the skeletal muscle to the HFD which is not maintained in the long run.

Among the strongest regulated genes we found five genes (*Dkk3, Hoxd8, Hoxd9, Hoxd10 *and *Tbx1*) which are especially known for their critical role during embryogenesis. The skeletal muscle is a dynamic tissue containing different types of stem cells such as satellite cells, side-population (SP) cells and muscle-derived stem cells (MDSC) [17,18]. To exclude the possibility that expression of these five embryogenesis-related genes originated from other cell types than myocytes we analyzed the expression of genes that specify satellite cells, SP cells and MDSC (satellite cells, *pax7*, *myf5*, *cd34 *and *c-met*; SP cells, *sca1*, *sdc4 *and *pax3*; MDSC, *cd34 *and *Blc2 *[17,18]). We found that only three genes (*cd34*, *sca1 *and *sdc4*) were detectably expressed (microarray signal intensity > 20) in the skeletal muscle of these mice. However, none of these detectably expressed markers differed more than 1.3-fold between the gastrocnemius and the quadriceps. Altogether, we cannot exclude the presence of cells other than myocytes, however, it is most unlikely that these cells have contributed to the different expression levels of *Dkk3, Hoxd8, Hoxd9, Hoxd10 *and *Tbx1*.

Two genes, *Dkk3 *and *Tbx1*, were expressed at a lower level in the gastrocnemius than in the quadriceps. Extrapolating our data to the soleus we found that *Dkk3 *expression in the soleus was lower than in the quadriceps or gastrocnemius. Interestingly, Tbx1 expression levels in the soleus were lower than in the quadriceps, but higher than in the gastrocnemius. During embryogenesis *Dkk3 *and *Tbx*1 are expressed in a variety of organs including the heart and limb buds [19,20]. Whereas the function of *Dkk3 *is still poorly understood [21], *Tbx1 *is well-studied especially in relation to heart development [20]. Recently it was shown that *Tbx1 *is also involved in development of the limb buds by regulating the number of myocytes [22]. Since further research revealed that *Tbx1 *is not necessary for skeletal muscle specification, differentiation, patterning or activation of the embryonic myogenic program, this is the only described function of *Tbx1 *in relation to embryonic muscle development [23]. Both *Dkk3 *and *Tbx1 *are reported to be detectably expressed in the adult skeletal muscle [19,24], but until now nothing is known about their possible function. We speculate that the relatively high expression levels of *Dkk3 *and *Tbx1 *in the quadriceps is involved in maintaining identity of this muscle type.

Three genes of the Hoxd gene cluster (*Hoxd8, Hoxd9 *and *Hoxd10*) were up-regulated in the gastrocnemius as compared with the quadriceps. The Hox gene family consists of at least 39 Hox genes organized in four gene clusters (A, B, C and D) which are subdivided into parallel groups numbered 1 to 13. The major function of Hox genes is the regulation of the formation of anterior-posterior patterning during embryonic development [25]. Different groups have studied the expression levels of the Hox gene family in adult tissues of human origin. They found that each tissue displays a unique combination of detectable Hox gene expression levels which is altered when normal cells changes to malignant cancer cells [26-28]. Thus, in addition to their critical role in embryonic development, Hox genes play an important role in adult cells by controlling critical processes like cellular identity and differentiation [29]. Houghton et al. [30]. have studied expression patterns of the Hox gene family in skeletal muscle tissue of adult mice. In line with our observations they reported that the *Hoxa10*, *Hoxc6, Hoxc9 *and *Hoxc10 *genes were detectably expressed. Furthermore, they could not detect *Hoxd11 *which also is consistent with our findings. Other members of the Hoxd gene cluster were not studied [30]. Thus to our knowledge, we are the first to show that *Hoxd8, Hoxd9 *and *Hoxd10 *are detectably expressed in adult murine muscle tissue, with highly increased expression levels in the gastrocnemius as compared with the quadriceps.

Together with the higher *Hoxd8 *and *Hoxd9 *gene expression levels in the gastrocnemius we found increased gene expression levels of markers for the more oxidative fiber types. Therefore we hypothesized that *Hoxd8 *and *Hoxd9 *might be involved in the determination of more oxidative muscle types. However, when we extrapolated our data to the soleus muscle we could not find a corresponding expression pattern between *Hoxd8, Hoxd9 *and *Myh7 *(marker for type I fibers) and *Tnni1 *(marker for type I fibers; data not shown). In fact, *Myh7 *and *Tnni1 *were highly expressed in the soleus, whereas *Hoxd8 *and *Hoxd9 *expression in the soleus was as low as in the quadriceps. Also the expression patterns of *Myh2, Myh1 *and *Myh4*, which are markers for type IIA, IIX and IIB fibers, respectively, did not show any resemblance with the expression pattern of *Hoxd8 *and *Hoxd9*. Although we did not find any resemblances between expression patterns of *Hoxd8*, *Hoxd9 *and any of the fiber type-specific markers we could distinguish the gastrocnemius, quadriceps and soleus from each other. Altogether, we suggest that the high expression levels of the *Hoxd8, Hoxd9 *and possibly *Hoxd10 *genes are a specific characteristic of the molecular profile of the gastrocnemius and are not related to an oxidative phenotype.

## Conclusion

We found that especially genes that are markers for oxidative fiber types and genes known to be important for embryogenesis were differentially expressed in the gastrocnemius vs. the quadriceps. The expression levels of these genes were not influenced by diet. Furthermore, expression of *Hoxd8 *and *Hoxd9 *was low and expression of *Dkk3 *and *Tbx1 *was high in the quadriceps. In the gastrocnemius we observed high expression of *Hoxd8 *and *Hoxd9 *and low expression of Dkk3 and Tbx1. Finally, in the soleus we found low expression of *Hoxd8, Hoxd9 *and *Dkk3*, but intermediate expression of *Tbx1*. Comparing gastrocnemius, quadriceps and soleus, the gene expression pattern of *Hoxd8 *and *Hoxd9 *did not correspond to the gene expression pattern of any of the fiber type-specific markers. Therefore, we conclude that the highly expressed embryonic genes *Dkk3, Hoxd8*, *Hoxd9 *and *Tbx1 *are involved in identifying muscle types in a diet-independent and fiber type-unrelated manner.

## Methods

### Mice and diet

Male C57BL/6J mice were obtained from Harlan (Horst, The Netherlands). At 9 weeks of age mice were switched to a run-in diet consisting of an LFD (10 kcal% fat) for 3 weeks. Following the run-in period mice were randomly assigned to the LFD or the HFD (45 kcal% fat). Both diets contained fat in the form of palm oil (based on D12450B and D12451; Research Diet Services, Wijk bij Duurstede, the Netherlands) as described previously [31].

#### Study 1

After 8 weeks of diet intervention, mice (n = 10 per diet) were fasted for 6 hours, anesthetized with a mixture of isofluorane (1.5%), nitrous oxide (70%) and oxygen (30%) and killed by cervical dislocation. Gastrocnemius and quadriceps muscles were dissected, snap-frozen in liquid nitrogen and stored at -80°C until further analysis.

#### Study 2

After 20 weeks of diet intervention, mice (n = 5 per diet) were fasted for 6 hour, anaesthetized and killed as described. Quadriceps, gastrocnemius and soleus muscles were dissected, snap-frozen in liquid nitrogen and stored at -80°C until further analysis.

Both animal studies were approved by the Local Committee for Care and Use of Laboratory Animals at Wageningen University.

### RNA isolation

Mouse total RNA was isolated from gastrocnemius, quadriceps and soleus muscles by mechanically homogenization in Trizol reagent (Invitrogen, Breda, the Netherlands). RNA was purified with the RNeasy Mini Kit (Qiagen, Venlo, the Netherlands) and contaminating genomic DNA was removed with the RNase-free DNase set (Qiagen). RNA quantity and purity was measured with the ND-1000 spectrophotometer (Isogen Life Science B.V., IJsselstein, the Netherlands). An A260/A280 ratio between 2.04 and 2.16 was found for all RNA samples. RNA integrity was checked on an Agilent 2100 BioAnalyzer (Agilent Technologies, Amsterdam, The Netherlands) using nanochips according to the manufacturer's instructions. All RNA samples showed an RIN value between 7.5 and 9.0.

### Affymetrix microarray analysis

RNA isolated from the gastrocnemius and quadriceps of mice fed an 8-week LFD or HFD was used for performing Affymetrix GeneChip^® ^Mouse Genome 430 2.0 arrays as described previously [14]. Array images were processed using packages from the Bioconductor project [32] and probe sets were redefined according to Dai et al. [33]. In this method probes are annotated using up-to-date databases and assigned to unique gene identifiers, in this case Entrez ID's, instead of the 'classic' GeneChip probe sets. This results in a less ambiguous and more accurate annotation. Arrays were normalized using quantile normalization, and expression estimates were calculated using GC-RMA, implementing the empirical Bayes estimate for non-specific binding [34]. Differentially expressed probe sets were identified using linear models, applying moderated t-statistics that implement empirical Bayes regularization of standard errors [35]. Comparisons were made between the gastrocnemius and quadriceps muscles using the fold change threshold method. In this method, most false-positives are caused by genes with low signal intensity and/or absent call (~50% of all genes). Thus, filtering out this constant noise before performing data analysis will improve the power to discriminate true changes from noise in the fold-change threshold method [36]. We manually set the signal threshold at 20, across all arrays, filtering out 60% of the genes. Probe sets that satisfied the criteria of a fold change > 1.3 and an FDR < 0.05, correcting for multiple-testing [37], were considered as differentially expressed.

To relate changes in gene expression to functional changes two complementary methods were used. The first method is based on overrepresentation of Gene Ontology (GO) terms, which uses a gene score resampling (GSR) method [38]. Full resampling was run with 200,000 iterations. Only classes with a false discovery rate (FDR) < 0.001 and with minimal 8 and maximal 125 genes were taken into account. The second method, gene set enrichment analysis (GSEA), is focused on predefined gene sets, that is, groups of genes that share biological function, chromosomal location or regulation [39]. The 'functional catalogue' constructed by Subramanian et al. was modified to contain only 505 well-defined murine, biochemical, metabolic and signal pathways compiled from the following publicly available, curated databases: BioCarta (BioCarta. BioCarta, 2005, p. http://www.biocarta.com.), GenMAPP [40], Kyoto Encyclopedia of Genes and Genomes (KEGG) [41], Sigma-Aldrich pathways (Sigma-Aldrich. Sigma-Aldrich Metabolic and Cell Signaling pathways, 2005.) and Signal Transduction Knowledge Environment (STKE; Signal Transduction Knowledge Environment. In: http://stke.sciencemag.org/. 2005.). The analysis was run using 1000 permutations per gene set. Gene sets with a FDR < 0.05 were considered as significantly regulated. The advantage is that both methods are unbiased and a score is computed based on all genes in a GO term or gene set.

The diet-sensitivity of differentially expressed genes, overrepresented GO classes and regulated gene sets was studied by comparing the gastrocnemius with the quadriceps under HFD conditions. Also in this comparison we used the criteria of a fold change > 1.3 and an FDR < 0.05. A heatmap of log-transformed microarray signal intensities was generated by using GeneMaths XT software (Sint-Martens-Latem, Belgium). Array data have been submitted to the Gene Expression Omnibus, accession number GSE18127.

### Quantitative real-time PCR for RNA

To validate microarray-detected changes as well as extrapolating microarray-detected changes to the gastrocnemius, quadriceps and soleus of 20-week LFD mice and HFD mice quantitative real-time PCR (qPCR) was performed using individual cDNA samples. RNA (1 μg in 20 μl) was reverse transcribed using the iScript cDNA synthesis kit (Biorad, Veenendaal, the Netherlands) containing RNase H+ iScript reverse transcriptase, a premixed RNase inhibitor to prevent indiscriminate degradation of RNA template, and a unique blend of oligo(dT) and random primers. cDNA was synthesized using a 3-step program (5 minutes (min) at 25°C, 30 min at 42°C and 5 min at 85°C). Primer sequences were retrieved from the online PrimerBank database [42], or otherwise designed using Beacon Designer5 (Biorad). Primers were tested for specificity by BLAST analysis. The qPCR reactions were performed in a volume of 25 μl containing 12.5 ng cDNA, 1× IQ SYBR Green Supermix (100 mM KCl, 40 mM Tris-HCI, 6 mM MgCl_2_, 0.4 mM of each dNTP, 50 units/ml iTaq DNA polymerase, SYBR Green I and 20 nM fluoresein; Biorad) and 400 nM of gene-specific forward and reverse primers (Biolegio, Nijmegen, the Netherlands). cDNA was amplified using a 2-step program (40 cycles of 10 seconds (sec) at 95°C and 45 sec at 60°C) with a MyiQ system (Biorad). Specificity of amplification was verified by melt curve analysis and evaluation of efficiency of PCR amplification. PCR reactions were performed in duplicate and gene expression levels were determined using iQ5 software (Biorad) using a Δ-Cq relative quantification model with PCR efficiency correction and multiple reference gene normalization [43,44]. As the *Canx, Hprt1 *and *Arbp *genes were identified as most stably expressed reference genes, we used the geometric mean of these three reference genes as normalization factor. Supplement 4 shows all relevant primer information.

### Statistical analyses

All data are expressed as means ± SE. Microarray data were analyzed as described above. All other statistical analyses were performed using Prism software (GraphPad Software, San Diego, CA, USA). Two-way ANOVA was used to test gene expression levels of Dkk3, *Hoxd8, Hoxd9, Myh1, Myh2, Myh4, Myh7 *and *Tbx1 *between muscle types and diets. When significant differences were found, a Bonferroni post hoc test was used to determine the exact location of the differences. A p value ≤ 0.05 was considered as statistically significant.

## Authors' contributions

JdW, ES and EM participated in the design and supervision of the study. JdW and MH performed the RNA isolations and qPCRs. PDG and MB assessed the quality control of the microarrays and provided support for microarray analysis. JDW performed microarray analysis and drafted the manuscript. EM and ES provided valuable feedback on the initial draft. All authors read and approved the final manuscript

## Supplementary Material

Additional file 1 **List of differentially expressed genes**. Additional file 1 gives the differentially expressed genes in the gastrocnemius as compared to the quadriceps from 10 LFD mice. To find these differentially expressed genes we used the criteria of an average signal intensity > 20 over all arrays, a fold change > 1.3 and an FDR < 0.05. Click here for file

Additional file 2 **Overview of qPCR analyses for 13 genes validating the microarray results**. Additional file 2 gives an overview of the microarray signal intensity, relative expression (qPCR), normalized expression (qPCR) and corresponding fold changes of 13 genes. Click here for file

Additional file 3 **Differentially expressed genes, overrepresented GO classes (ErmineJ) and regulated gene sets (GSEA) between gastrocnemius and quadriceps in either LFD or HFD conditions**. Additional file 3 gives an overview of the differentially expressed genes, overrepresented GO classes (ErmineJ) and regulated gene sets (GSEA) in the gastrocnemius as compared to the quadriceps in HFD and LFD conditions. Click here for file

Additional file 4 **Primer information**. Additional file 4 gives the sequences, start, end and amplicon length of the used primer pairs. Click here for file
